# Novel Chromone-Containing Allylmorpholines Induce Anxiolytic-like and Sedative Effects in Adult Zebrafish

**DOI:** 10.3390/biomedicines10112783

**Published:** 2022-11-02

**Authors:** Veronika A. Prikhodko, Yuri I. Sysoev, Elena V. Gerasimova, Sergey V. Okovityi

**Affiliations:** 1 Department of Pharmacology and Clinical Pharmacology, Saint Petersburg State Chemical and Pharmaceutical University, 197376 Saint Petersburg, Russia; 2 Laboratory of Targeted Intra-Brain Drug Delivery, N.P. Bechtereva Institute of the Human Brain of the Russian Academy of Sciences, 197376 Saint Petersburg, Russia; 3 Laboratory of Neuromodulation of Motor and Visceral Functions, I.P. Pavlov Institute of Physiology of the Russian Academy of Sciences, 199034 Saint Petersburg, Russia; 4 Department of Neurobiology, Sirius University of Science and Technology, 353340 Sochi, Russia; 5 Institute of Translational Biomedicine, Saint Petersburg State University, 199034 Saint Petersburg, Russia

**Keywords:** allylmorpholines, chromone derivatives, morpholine derivatives, sedatives, anxiolytics, acetylcholinesterase, NMDA receptors, zebrafish, behavioral testing, novel tank test

## Abstract

Chromone-containing allylmorpholines (CCAMs) are a novel class of compounds that have demonstrated acetyl- and butyryl-cholinesterase-inhibiting and N-methyl-D-aspartate (NMDA) receptor-blocking properties in vitro, but their in vivo pharmacological activity remains underexplored. In this work, we evaluated the psychotropic activity of five different CCAMs (1 (9a), 2 (9j), 3 (9l), 4 (33a), and 5 (33b)) using the novel tank test (NTT) and light/dark box (LDB) test in adult zebrafish. The CCAMs were screened in the NTT at a range of concentrations, and they were found to induce a dose-dependent sedative effect. Compound **4** (**33a**) was also evaluated using the LDB test, and it was found to have anxiolytic-like properties at low concentrations. To assess the potential contribution of the glutamate and cholinergic mechanisms in the effects of the CCAMs, we conducted experiments with pre-exposure to putative antagonists, NMDA and biperiden. Neither biperiden nor NMDA were able to diminish or cancel the effects of the CCAMs, countering the in vitro data obtained in previous studies. The apparent discrepancy could be related to the specifics of CCAM metabolism or to the interspecies differences between the putative target proteins, possibly due to the relatively low identity percentage of their sequences. Although further research in mammals is required in order to establish their pharmacological properties, novel CCAMs may represent an appealing group of psychoactive drug candidates.

## 1. Introduction

The course of the preclinical development of novel neuro- or psycho-active agents involves the choice of a relevant lead compound class and the choice of a relevant animal model [1]. Leads may be searched for among compounds that occur naturally, that are products of directed microbial biosynthesis, or that are obtained through chemical synthesis. Morpholine derivatives, an example of the latter, appear to be a promising compound class for drug discovery and development, as they are highly versatile and exhibit an extensive spectrum of biological activity.

Morpholine (1,4-tetrahydrooxazine, or 1-oxa-4-azacyclohexane) is a six-membered aliphatic heterocyclic compound with the chemical formula O(CH_2_CH_2_)_2_NH. Because of their versatile physicochemical properties and high availability, morpholine derivatives are widely used in organic chemistry as bases, catalysts, ligands, chiral auxiliaries, surface-active agents, solvents, and synthetic building blocks. For example, functionalized morpholines are commonly employed as precursors for the synthesis of enantiomerically pure α-amino acids, β-amino alcohols, peptides, and other biologically active substances [2].

Morpholine is not known to occur freely in nature, and it is considered to be a relatively rare moiety in natural compounds. However, several morpholine-containing alkaloids isolated from plants and marine fauna have been shown to possess antimicrobial, anti-inflammatory, antioxidant, and antitumor properties [1,3], and a morpholine-conjugated phenethyl alcohol derivative found in American cockroaches has been found to exhibit antiangiogenic activity in vitro [4]. Morpholine rings are much more common in synthetic pharmaceuticals, including β-blockers (timolol); antiarrhythmics (moracizine); nonsteroidal anti-inflammatory drugs (morazone, emorfazone, and filenadol); anticoagulants (rivaroxaban); immunosuppressants (mycophenolate mofetil); antitumor (gefitinib and canertinib), antibacterial (linezolid, linopristin, levofloxacin, ofloxacin, and finafloxacin), and antifungal (amorolfine) agents; and pharmacoenhancers (cobicistat) [5]. Antisense phosphorodiamidate morpholino oligonucleotides, such as eteplirsen, golodirsen, viltolarsen, and casimersen, represent a newer class of physiologically active compounds with potential applications in gene therapy, as well as in fundamental biology research [6].

More recently, morpholine has attracted increased attention as a pharmacophoric element, scaffold structure, and pharmacoenhancer, with particular relevance to the design and optimization of central nervous system agents [3,7]. The morpholine nucleus facilitates drug transport across the blood–brain barrier, and it plays a key role in establishing molecular interactions with an extensive variety of central enzymatic and receptor targets [6,7]. Monoamine oxidases A and B, cholinesterases, β/γ/δ-secretases, leucine-rich repeat kinase 2, adenosine kinase, cannabinoid (CB1/2), acetylcholine (α_7_-H), serotonin (5-HT_1A/B_, 5-HT_2A_, 5-HT_3_), dopamine (D_4_), histamine (H3), glutamate (mGlu_2_, N-methyl-D-aspartate (NMDAR), AMPAR), γ-aminobutyric acid (GABA), adenosine (A_2A_), sigma (σ_1/2_), neurokinin (NK_1_) receptors, and nuclear factor erythroid 2-related factor 2 (Nrf2) have to date been identified as the major central targets of morpholine derivatives [7,8,9,10,11]. 

Known centrally acting morpholine-containing compounds include stimulants/anorectics (phenmetrazine and analogues); dissociative anesthetics (phencyclidine analogues); anticonvulsants (imepitoin); antiemetics (azasetron, aprepitant, and analogues); antidepressants (reboxetine, moclobemide, and viloxazine); tranquilizers (trimetozine); antipsychotics (molindone); central analgesics (pravadoline and analogues); opioids (dextromoramide, morpheridine, phenadoxone, and pholcodine); non-opioid central antitussives (morclofone and pentethylcyclanone); and antiparkinsonian (foliglurax and tozadenant), neuroprotective (WIN 55,212-2 and PRE-084), and nootropic (CX717 and A-349,821) agents [5]. Potential indications for morpholine-based drugs may, in turn, include Parkinson’s disease, Alzheimer’s disease, Huntington’s disease, multiple sclerosis, lateral amyotrophic sclerosis, traumatic brain injury, and ischemic stroke [12].

In 2018, Chernov et al. reported the synthesis of a series of novel chromone-containing allylmorpholines (CCAMs) demonstrating acetyl- and butyryl-cholinesterase (AChE and BChE)-inhibiting and NMDAR-blocking properties in vitro [13]. As both AChE and NMDAR have long been considered viable therapeutic targets for neuroprotection and cognitive enhancement [14,15,16,17], CCAMs appear to be a promising group of drug candidates for the treatment of neurological disorders. In a recent study, a novel CCAM was found to exert sedation in rats with traumatic brain injury [18]. However, to the best of our knowledge, no in vivo screening of this class of compounds’ behavioral activity has been performed to date.

The successful development of a psychoactive drug candidate requires the choice of an animal model that is suitable, relevant, and effective [1]. The zebrafish (*Danio rerio* HAMILTON) is an emerging biological model system commonly used in experimental pharmacology, toxicology, neuroscience, and behavior research. Zebrafish are characterized by faster development, a longer lifespan, and a higher reproductive rate than rodents, and they are fairly easy to maintain and handle in laboratory conditions. 

Several major neurotransmitter systems, including the cholinergic and glutamatergic systems, have been confirmed to be present in the zebrafish central nervous system, and they have high molecular and structural homologies to humans [19,20]. Adult zebrafish exhibit behavioral phenotypes observed in mammals, and they are sensitive to virtually all classes of neurotropic drugs, which makes them an appealing model organism for translational neuropharmacology [21,22]. The translational value of the zebrafish is supported by the fact that approximately 70% of human genes have at least one zebrafish orthologue [23].

The novel tank test (NTT), which is conceptually similar to the rodent open-field test, is the most commonly used behavioral paradigm in adult zebrafish, and it exploits the natural behavior of zebrafish whereby they seek protection in an unfamiliar environment by diving to the bottom and remaining there until they feel safe enough to explore [24]. In view of the above, this study was conducted to evaluate the effects of different CCAMs on zebrafish behavior using the NTT.

## 2. Materials and Methods

### 2.1. Animals and Drugs

A total of 672 wild-type short-fin adult zebrafish *Danio rerio* (~50:50 male:female ratio, 4–6 months old) were obtained from a local commercial supplier (Aksolotl Ltd., St. Petersburg, Russia) and kept for at least two weeks to acclimate to the holding facility. All animals used in the present study belonged to the same batch, and they were housed in 50 L thermostatic tanks filled with aerated, filtered, non-chlorinated water at 27 ± 1 °C, pH 7.0–7.2 [25,26]. Fluorescent ceiling-mounted light tubes provided room illumination under a 14/10 light/dark photoperiod cycle (lights on at 7:00 a.m. and off at 9:00 p.m.) [27]. The fish were fed thrice daily with commercial flake food TetraMin Pro (Tetra GmbH, Osnabrück, Germany). Animal experiments were approved by the Institutional Animal Care and Use Committee, and they fully adhered to the National and Institutional guidelines and regulations on animal experimentation. All animals tested were included in the final analyses, without removing outliers. All experiments were performed as planned, and all analyses and endpoints assessed were included without omission. The CCAMs were obtained from the Department of Organic Chemistry of the St. Petersburg State Chemical and Pharmaceutical University (St. Petersburg, Russia).

A total of 672 wild-type short-fin adult zebrafish *Danio rerio* (~50:50 male:female ratio, 4–6 months old) were obtained from a local commercial supplier (Aksolotl Ltd., St. Petersburg, Russia) and kept for at least two weeks to acclimate to the holding facility. All animals used in the present study belonged to the same batch, and they were housed in 50 L thermostatic tanks filled with aerated, filtered, non-chlorinated water at 27 ± 1 °C, pH 7.0–7.2 [25,26]. Fluorescent ceiling-mounted light tubes provided room illumination under a 14/10 light/dark photoperiod cycle (lights on at 7:00 a.m. and off at 9:00 p.m.) [27]. The fish were fed thrice daily with commercial flake food TetraMin Pro (Tetra GmbH, Osnabrück, Germany). Animal experiments were approved by the Institutional Animal Care and Use Committee, and they fully adhered to the National and Institutional guidelines and regulations on animal experimentation. All animals tested were included in the final analyses, without removing outliers. All experiments were performed as planned, and all analyses and endpoints assessed were included without omission. The CCAMs were obtained from the Department of Organic Chemistry of the St. Petersburg State Chemical and Pharmaceutical University (St. Petersburg, Russia).

### 2.2. Experimental Design and Behavioral Testing

**Figure 1:**
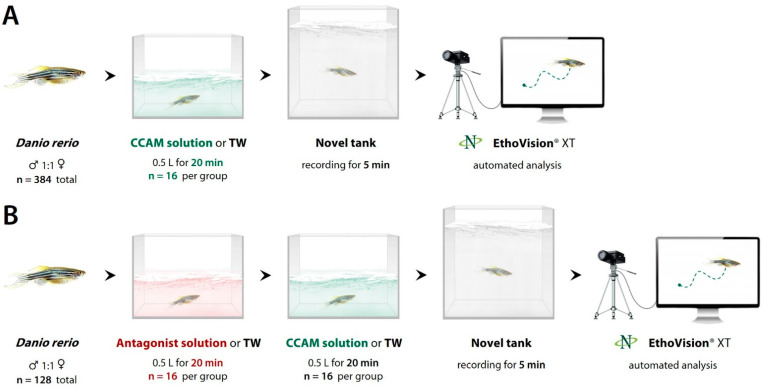
Design of Experiments 1–5 and 8 (**A**) and Experiments 6 and 7 (**B**). CCAM, chromone-containing allylmorpholine; TW, tank water.

Behavioral testing was performed between 12:00 p.m. and 5:00 p.m. [28]. The fish were randomly assigned into several experimental groups (*n* = 16 fish per group). Experiments 1–5 were conducted to evaluate the effects of five different CCAMs (1–5 (Table 1)) in a wide concentration range on zebrafish behavior in the NTT according to the experimental design illustrated in Figure 1A.

Prior to testing, the fish were individually placed for 20 min into small 0.5 L opaque plastic containers filled with tank water or aqueous CCAM solutions of given concentrations, with the temperature adjusted to the holding room temperature. Following acute exposure, the fish were individually transferred to the NTT in order to assess their locomotion and behavior over a 5 min period. The NTT (20 × 20 × 5 cm, made of plexiglass; Open Science, Krasnogorsk, Russia) was divided by an imaginary marker line into top and bottom zones of equal area [23,29]. Trials were recorded with a CNE-CWC1 web camera (Canyon, ASBIS, Limassol, Cyprus) for further analyses, and then they were processed offline using EthoVision XT 11.5 software (Noldus IT, Wageningen, Netherlands) to calculate the total distance traveled (cm), mean and maximum velocity (cm/s), number of freezes (velocity < 2 cm/s), total freezing duration (s), total time spent in the top zone (s), number of entries into the top zone, and latency of the first bottom–top transition (s) [23,29,30,31,32].

Because we observed possible anxiolytic effects of low concentrations of compound **4**, Experiment 4a was conducted to evaluate an even lower range of concentrations, 0.5–5 mg·L^−1^. Experiments 6, 7, and 8 were conducted to determine whether the effects of the CCAMs were due to NMDAR antagonism and would therefore be reduced or completely prevented by NMDA itself (Sigma-Aldrich, St. Louis, MO, USA). Compound **1** was chosen for these experiments since it was found to potently modulate zebrafish behavior in Experiment 1. In Experiment 6, the fish were pre-exposed for 20 min to a 3.83 mg·L^−1^ aqueous NMDA solution (equimolar to 20 mg·L^−1^ compound **1**) (or tank water for control) in a plastic container, and then they were transferred to an identical container filled with an equivolume compound **1** solution (or tank water for control) and tested as described above (Figure 1B). In Experiment 7, we tested whether NMDA would act as a psychostimulant in zebrafish at a higher concentration of 100 mg·L^−1^, using a design identical to that of Experiments 1–5. 

Additionally, to investigate the role of NMDAR in the modulation of locomotion and anxiety-like behavior in zebrafish, Experiment 8 was conducted using quinolinic acid (Sigma-Aldrich, USA), a potent NMDAR agonist [33,34], at a concentration range according to the same design (Figure 1A). In order to explore the potential role of AChE/BChE inhibition in the central effects of the CCAMs, we conducted Experiment 9 similarly to Experiment 6, using an equimolar (8.11 mg·L^−1^) aqueous biperiden solution (Akineton^®^, Desma GmbH, Mainz-Kastel, Germany/Laboratorio Pharmaceutico SIT, Mede, Italy) instead of NMDA (Figure 1B). 

Finally, to further explore the psychotropic activity profile and to assess the anxiety-modulating properties of compound **4**, Experiments 10 and 11 were conducted using the NTT and the light/dark box (LDB) test, respectively. Experiment 10 was conducted according to a design identical to that of Experiments 1–5, using compound **33a** at the concentrations of 0.5, 1, and 5 mg·L^−1^. 

The LDB test utilizes the fish’s scototaxis (aversion to bright areas and natural preference for the dark), and it can be used to assess anxiety-like behavior in zebrafish [35], as well as in rodents [24,30,36,37]. Zebrafish were exposed for 20 min to either tank water or an aqueous solution of compound **4** at low concentrations of 0.5, 1, or 5 mg·L^−1^, and then they were transferred into an LDB testing environment (Open Science, Russia). Fish behavior was recorded using a camera for 5 min and analyzed using RealTimer 1.15 software (Open Science, Russia) to calculate the latency of the first entry into the light zone (s), total number of zone transitions, and total time spent in the light zone (s). The working concentrations of all agents used in this study are given in Table 2.

### 2.3. Sequence Identity Analysis

Sequence identity was quantified using the nucleotide and protein basic local alignment search tool (BLAST) provided by National Center for Biotechnology Information (NCBI) [38,39]. Gene and protein sequences for *Mus musculus*, *Rattus norvegicus*, and *D. rerio* were obtained from the NCBI Gene database [40] and heat-mapped based on their fraction identity to *Homo sapiens* homologous sequences. For gene polymorphisms and protein isoforms, fraction identity means were mapped. Protein structures were obtained from the Research Collaboratory for Structural Bioinformatics Protein Data Bank (RSCB PDB) [41] and processed using USCF Chimera 1.11.2 software [42].

## 3. Results

### 3.1. CCAM Screening

#### 3.1.1. Sedative Activity of Compound **1** (**9a**) in the Novel Tank Test

Compound **1** at 10 and 20 mg·L^−1^ reduced the distance covered, the mean velocity, and the number of bottom-to-top transitions (*p* < 0.01 vs. control for all), and it increased the total freezing duration (*p* < 0.01 vs. control for 10 mg·L^−1^; *p* < 0.05 vs. control for 20 mg·L^−1^). In addition, at 20 mg·L^−1^, compound **1** increased the latency to the first transition into the top zone compared to the control (*p* < 0.05). The lowest concentration, 1 mg·L^−1^, appeared to be much less potent (*p* < 0.05, *p* < 0.01), as it was only able to decrease the time the zebrafish spent in the top zone of the NTT (*p* < 0.05 vs. control). 

**Figure 2:**
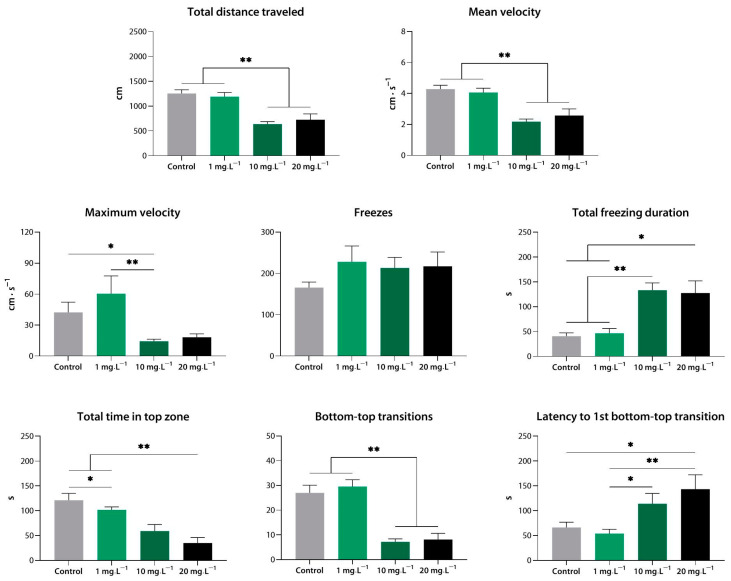
Compound **1** (**9a**) induces sedation in zebrafish at a range of concentrations, as assessed using the novel tank test. *, *p* < 0.05; **, *p* < 0.01.

Notably, a clear dose-dependent pharmacological response was observed within the working concentration range (Figure 2). We were unable to test compound **1** at 100 mg·L^−1^ due to its toxicity.

#### 3.1.2. Sedative Activity of Compound **2** (**9j**) in the Novel Tank Test

**Figure 3:**
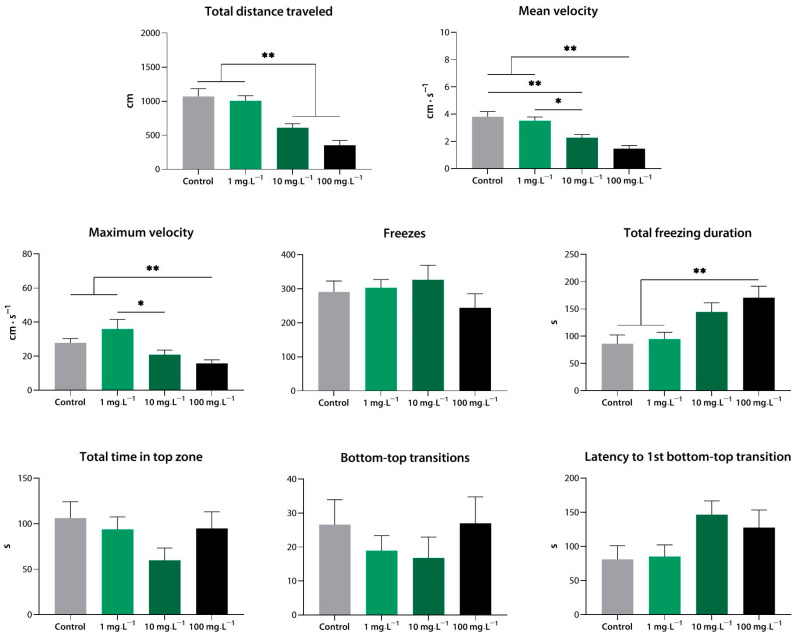
Compound **2** (**9j**) induces sedation in zebrafish at a range of concentrations, as assessed using the novel tank test. *, *p* < 0.05; **, *p* < 0.01.

Compound **2** at 100 mg·L^−1^ markedly reduced the total distance covered, mean and maximum velocity, and total freezing duration (*p* < 0.01 vs. control). At 10 mg·L^−1^, it also decreased the mean velocity of the zebrafish (*p* < 0.01 vs. control). As with compound **1**, the concentration of 1 mg·L^−1^ appeared to be too low to have detectable effects on zebrafish behavior in the NTT (*p* < 0.05, *p* < 0.01 vs. 10 and 100 mg·L^−1^) (Figure 3).

#### 3.1.3. Sedative Activity of Compound **3** (**9l**) in the Novel Tank Test

**Figure 4:**
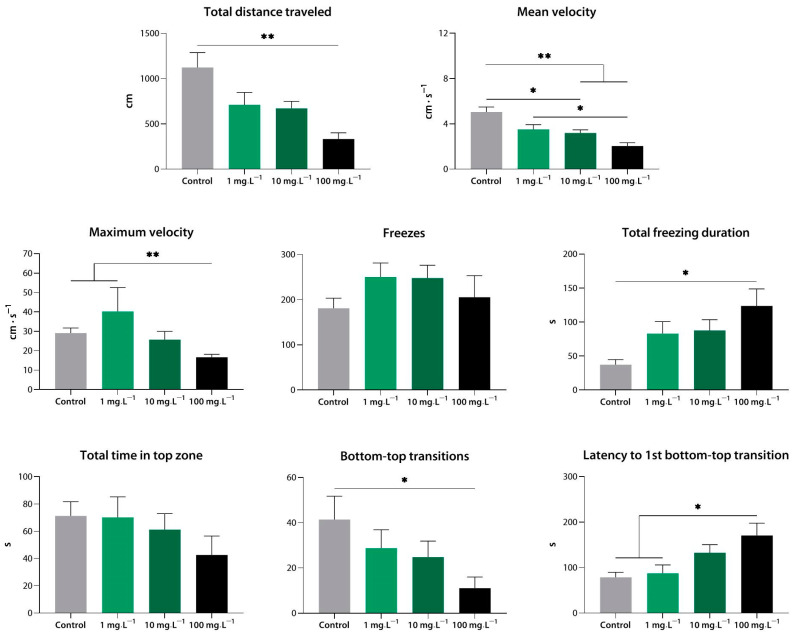
Compound **1** (**9l**) induces sedation in zebrafish at a range of concentrations, as assessed using the novel tank test. *, *p* < 0.05; **, *p* < 0.01.

Exhibiting a similar activity profile, compound **3** at 100 mg·L^−1^ reduced the total distance traveled, the mean and maximum velocity, and the number of transitions, and it increased the total freezing duration, as well as the latency of the first transition into the top zone compared to the control values (*p* < 0.05, *p* < 0.01). Compound **3** at 10 mg·L^−1^ reduced the mean velocity (*p* < 0.05 vs. control), while the lowest concentration failed to exert any effects of its own and was significantly weaker than 10 and 100 mg·L^−1^ (*p* < 0.05, *p* < 0.01) (Figure 4).

#### 3.1.4. Sedative Activity of Compound **4** (**33a**) in the Novel Tank Test

**Figure 5:**
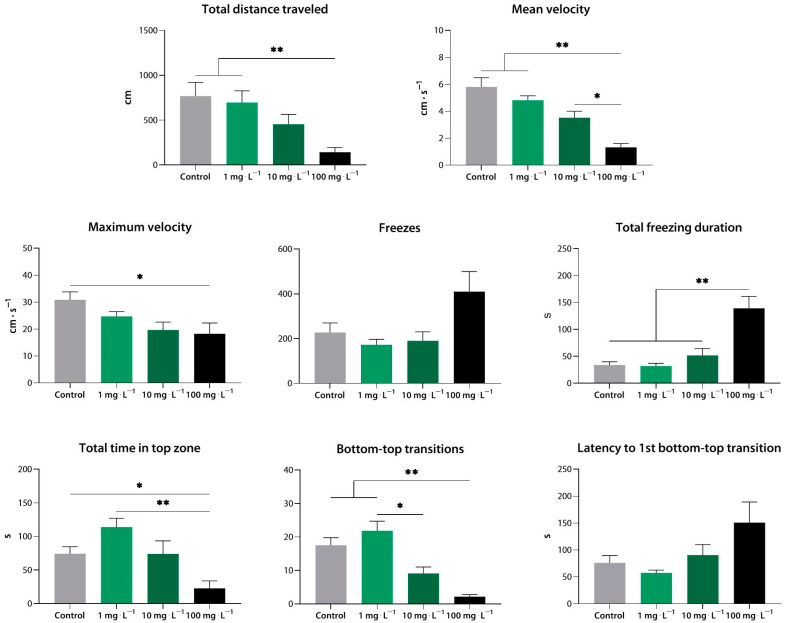
Compound **4** (**33a**) induces sedation in zebrafish at a range of concentrations, as assessed using the novel tank test. *, *p* < 0.05; **, *p* < 0.01.

At 100 mg·L^−1^, compound **4** reduced the total distance, the mean and maximum velocity, the total time spent in the top zone of the NTT, and the number of zone transitions (*p* < 0.05, *p* < 0.01 vs. control). At 1 and 10 mg·L^−1^, it exhibited lower efficacy and was unable to significantly affect zebrafish behavior. The lowest concentration, 1 mg·L^−1^, appeared to be inferior to the higher ones when measuring the total time spent in the top zone (*p* < 0.01 vs. 100 mg·L^−1^) and the number of transitions (*p* < 0.01 vs. 10 mg·L^−1^) (Figure 5). There was a slight tendency of lower (1–10 10 mg·L^−1^) concentrations to increase the number of transitions into the top zone, as well as the time spent in it, indicating potential anxiolytic activity, which was evaluated in Experiments 10 and 11.

#### 3.1.5. Sedative Effect of Compound **5** (**33b**) in the Novel Tank Test

**Figure 6:**
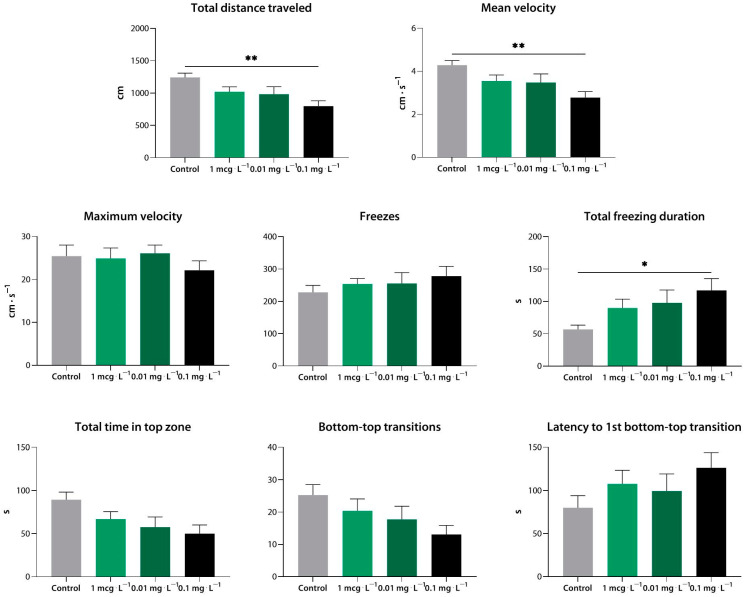
Compound **5** (**33b**) induces sedation in zebrafish at a range of concentrations, as assessed using the novel tank test. *, *p* < 0.05; **, *p* < 0.01.

Compound **5** was tested at the low concentrations of 0.001, 0.01, and 0.1 mg·L^−1^, as it has previously been reported to have potent activity in vitro [11]. At 0.1 mg·L^−1^, it markedly reduced the distance traveled and mean velocity of the zebrafish (*p* < 0.01 vs. control), and it increased the total freezing duration (*p* < 0.05 vs. control). At lower concentrations, no significant effect was observed (Figure 6).

### 3.2. Putative Antagonist Testing

#### 3.2.1. Effects of Compound **1** (**9a**) Following Equimolar N-Methyl-D-Aspartate Pre-Exposure in the Novel Tank Test

As expected based on the Experiment 1 results, compound **1** markedly decreased the total distance traveled, the mean and maximum velocity, and the number of transitions while at the same time increasing the freezing frequency (*p* < 0.01 for all) and duration (*p* < 0.05 vs. control). 

**Figure 7:**
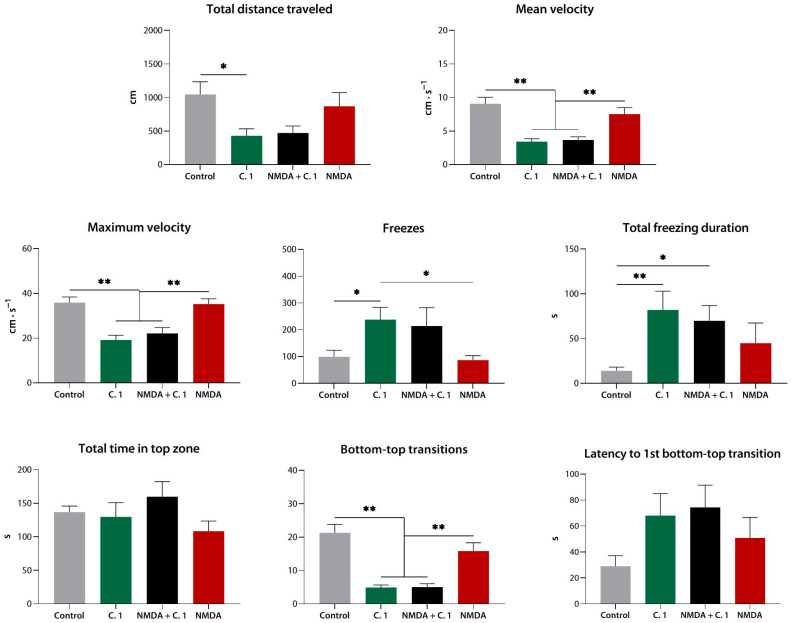
Equimolar N-methyl-D-aspartate (NMDA) pre-exposure does not diminish or cancel the sedative effect of compound **1** (**9a**) in the novel tank test. C. 1, compound **1**; *, *p* < 0.05; **, *p* < 0.01.

NMDA, however, at a concentration equimolar to 20 mg·L^−1^ compound **1**, did not produce any detectable effect on zebrafish behavior compared to control. No apparent antagonism between the two agents was observed, as NMDA pre-exposure did not reduce or in any other way alter the effects of compound **1**. Moreover, no differences were detected between the compound **1** and NMDA + compound **1** groups, indicating the absence of the modulation of compound **1** effects by NMDA (Figure 7).

#### 3.2.2. Effects of N-Methyl-D-Aspartate in the Novel Tank Test

**Figure 8:**
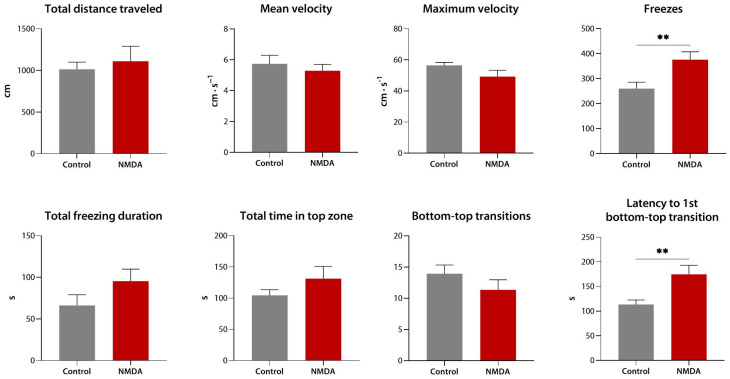
N-methyl-D-aspartate (NMDA) at 100 mg·L^−1^ does not induce a psychostimulant-like effect in zebrafish, as assessed using the novel tank test. **, *p* < 0.01.

Surprisingly, NMDA itself, at a much higher dose of 100 mg·L^−1^, increased the freezing frequency and latency to enter the top zone (*p* < 0.01 for both), but it had little to no effect on the other zebrafish behavior parameters (Figure 8).

#### 3.2.3. Sedative Effect of Quinolinic Acid in the Novel Tank Test

**Figure 9:**
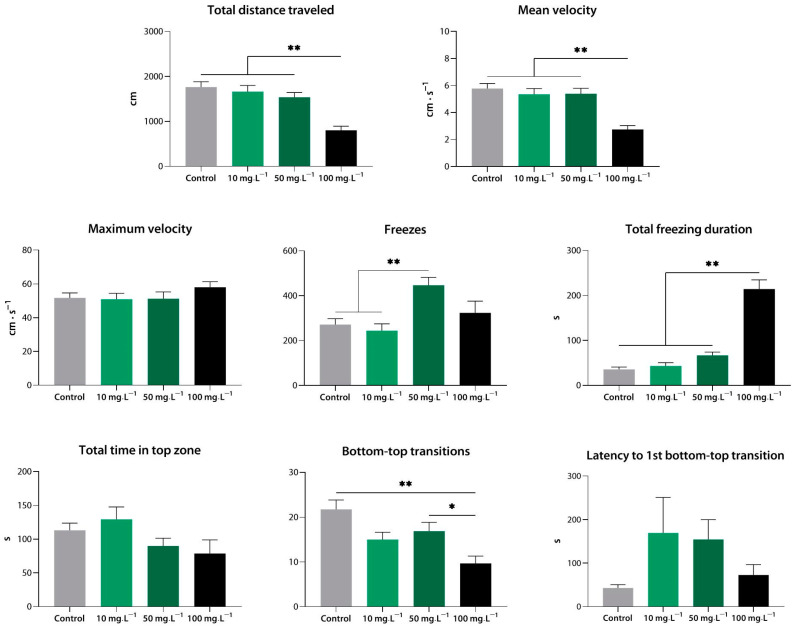
Quinolinic acid induces sedation in zebrafish at a range of concentrations, as assessed using the novel tank test. *, *p* < 0.05; **, *p* < 0.01.

Quinolinic acid reduced the total distance traveled, the mean velocity, and the number of bottom-to-top transitions at the highest concentration, 100 mg·L^−1^, while at the same time increasing the number of freezes at 50 mg·L^−1^ and the total freezing duration at 100 mg·L^−1^ (*p* < 0.01 for all). Of note, a clear positive exposure–response relationship was observed when several of the parameters were measured, namely, distance traveled, mean velocity, and freezing duration, suggesting a dose-dependent response (Figure 9).

#### 3.2.4. Effects of Compound **1** (**9a**) Following Equimolar Biperiden Pre-Exposure in the Novel Tank Test

**Figure 10:**
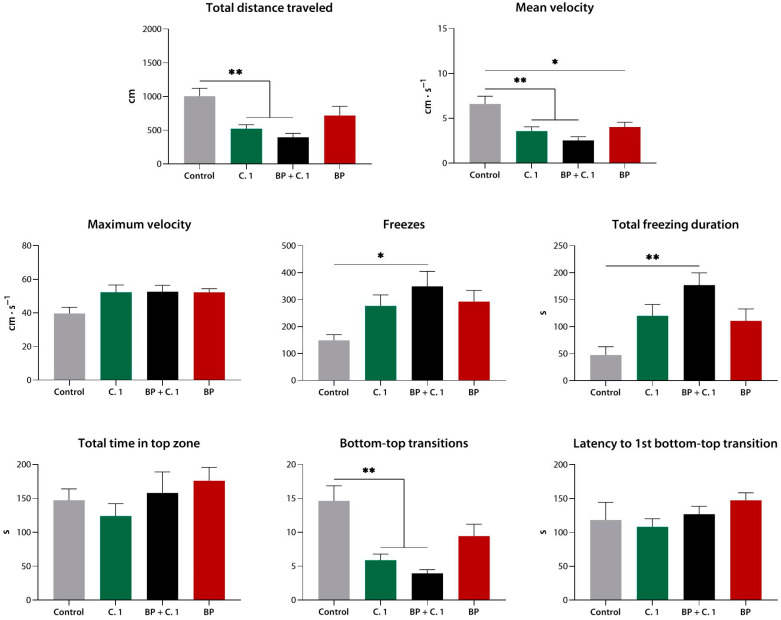
Equimolar biperiden pre-exposure does not diminish or cancel the sedative effect of compound **1** (**9a**) in the novel tank test. C. 1, compound **1**; *, *p* < 0.05; **, *p* < 0.01.

Compared to the control, compound **1** reduced the distance covered, the mean velocity, and the number of bottom-to-top transitions (*p* < 0.01 for all), consistent with its effects observed in Experiment 1. Biperiden alone modestly reduced the mean velocity of the fish (*p* < 0.05) but lacked any other behavioral effects. When compound **1** was given after a 20 min pre-exposure to biperiden, a slight synergistic relationship between the two agents was observed, namely, in the frequency and duration of freezing (*p* < 0.01, *p* < 0.05 vs. control, respectively). However, no significant differences from either compound **1** or biperiden alone were observed in the combination group (Figure 10).

### 3.3. Anxiolytic-like Activity of Compound **33a**

#### 3.3.1. Anxiolytic-like Effect of Compound **4** (**33a**) in the Novel Tank Test

**Figure 11:**
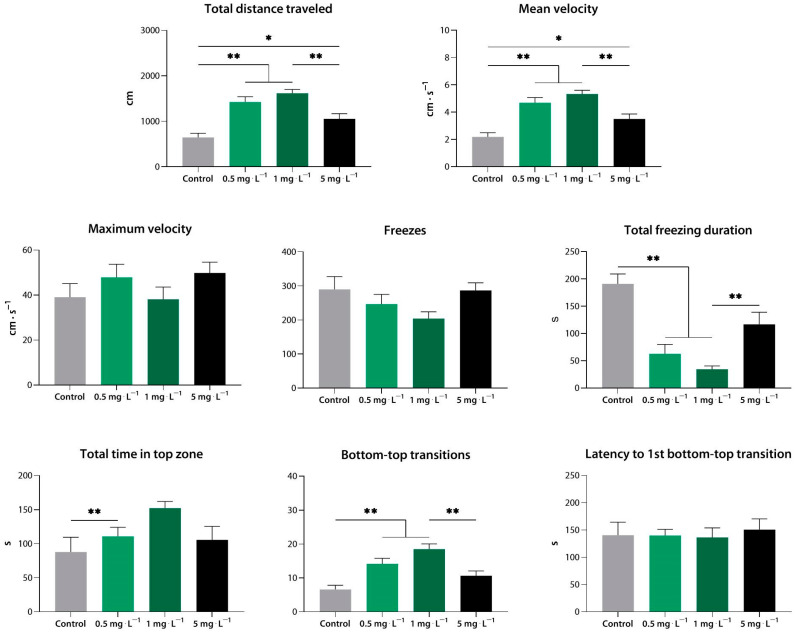
Compound **4** (**33a**) at low concentrations induces an anxiolytic-like effect in zebrafish, as assessed using the novel tank test. *, *p* < 0.05; **, *p* < 0.01.

At 0.5–5 mg·L^−1^, compound **4** increased the total distance, the mean velocity, the time spent in the top zone, and the number of transitions, and it reduced the freezing duration (*p* < 0.01, *p* < 0.05 vs. control). Notably, 5 mg·L^−1^ appeared to be less effective than 1 mg·L^−1^ when measuring the distance traveled, mean velocity, freezing duration, and transition frequency (*p* < 0.01 vs. 1 mg·L^−1^ for all) (Figure 11).

#### 3.3.2. Anxiolytic-like Effect of Compound **4** (**33a**) in the Light/Dark Box Test

**Figure 12:**
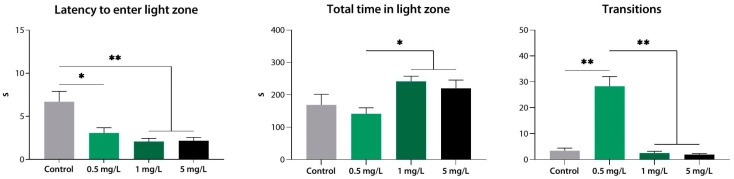
Compound **4** (**33a**) at low concentrations induces an anxiolytic-like effect in zebrafish, as assessed using the light/dark box test. *, *p* < 0.05; **, *p* < 0.01.

In the LDB test, low concentrations of compound **4** markedly reduced the latency of the first entry into the light zone compared to the control group (*p* < 0.05 for 0.5 mg·L^−1^; *p* < 0.01 for 1 and 5 mg·L^−1^), while 0.5 mg·L^−1^ also increased the number of transitions between the zones (*p* < 0.01). At 1 and 5 mg·L^−1^, compound **4** caused a greater increase in the total time spent in the light zone than did 0.5 mg·L^−1^ (*p* < 0.05) while at the same time having no apparent effect on the number of transitions (*p* < 0.01 vs. 0.5 mg·L^−1^) (Figure 12).

## 4. Discussion

### 4.1. CCAM Screening

In the NTT, all CCAMs reduced the total distance covered by the fish and the mean velocity, and they increased the total freezing duration, with most of the compounds also reducing the maximum velocity and the number of bottom-to-top transitions (*p* < 0.05, *p* < 0.01). Such effects in the NTT are typical of sedative agents, as has been previously confirmed by numerous studies of well-known sedatives, e.g., diazepam [43], chlordiazepoxide [44], clonazepam [45], and high-dose ethanol [46]. 

Compound **5** has been previously evaluated as a potential neuroprotecting agent in rats with unilateral traumatic brain injury; however, it failed to demonstrate any neuroprotective activity. Nonetheless, it induced dose-dependent sedation in rats [18] and in zebrafish in our experiment. Notably, the CCAMs exerted a sedative effect over a wide range of concentrations; this could be related not only to their different inherent activities shown in vitro [13] but also to their varying solubility in water, which would affect their availability and the exposure of zebrafish to them. 

There is a considerable possibility that the dose-dependent decrease in zebrafish locomotion did not result from the general toxicity of the CCAMs, since one of the molecules, 33a, has been found to possess anxiolytic activity at lower concentrations in both the NTT and the LDB test. Biphasic anxiolytic and sedative activities at lower and higher doses, respectively, are characteristics of such agents as ethanol [46], benzodiazepines, barbiturates, and gabapentinoids [47]. Moreover, the CCAMs were used at concentrations several times lower than their median lethal doses (compound **5** (**33b**): LD_50_ = 320 mg·kg^−1^ b.w., mouse, intraperitoneally; LD_50_ > 1000 mg·kg^−1^ b.w., mouse, orally (data not published)). 

In view of the above, we can hypothesize that the CCAMs themselves or their metabolites may be able to cross the blood–brain barrier (BBB) in adult zebrafish and exert neurobehavioral effects. However, the probability of metabolite generation within such a short time frame (20 min) appears to be low, as it would require a very fast metabolism rate for the CCAMs. In conclusion, further research is needed to establish whether the CCAMs and/or their metabolites can cross the BBB, as well as to completely exclude the possibility of toxicity being the reason for the observed changes in zebrafish behavior following exposure to the CCAMs.

### 4.2. Contribution of Glutamatergic and Cholinergic Mechanisms to the Effects of the CCAM

Biperiden and NMDA showed no antagonistic activity towards compound **9a** in Experiments 6 and 9, which appears to be inconsistent with the results of in vitro screening that suggested anticholinergic and NMDA-blocking mechanisms in the CCAMs [13].

On the one hand, this could indicate the presence of other mechanisms mediating the pharmacological activity of the CCAMs in vivo, which does not have to be in direct conflict with the data obtained in vitro. For example, these compounds could have active metabolites with different structures and distinct pharmacological properties, directly responsible for the central effects of the CCAMs. This would not be unusual for a neuropharmacological study, as such is the case with opiates and opioids [48], carbamazepine [49], primidone [50], and many others. 

On the other hand, the effects of the NMDAR agonists NMDA and quinolinic acid, namely, the relatively low activity of the former and the dose-dependent sedation caused by the latter, were quite surprising. Supposedly, NMDAR activation produced more complex changes in zebrafish behavior in the NTT rather than a simple increase or decrease in locomotion. For example, the NMDAR antagonist ketamine has been shown to induce hyperactivity and aggression at sub-anesthetic concentrations (2 mg·mL^−1^), while higher concentrations (20–40 mg·mL^−1^), in turn, have been shown to suppress aggression and other psychotic-like symptoms [51]. However, tiletamine, a close analogue of ketamine, has been found to exert robust dose-dependent sedation at 5–10 mg·mL^−1^ [52]. The MK-801 sensitivity in zebrafish appears to be lower than that in mammals, and it is age-dependent, which could be a consequence of differential NMDA receptor subunit expression [53].

The zebrafish is known to share fair gene sequence identity with *Homo sapiens*; however, a significant phylogenetic distance still exists, as the similarity between the two species’ genomes only amounts to ~70% [54]. Glutamatergic circuits and glutamate, as well as muscarinic cholinergic receptors and acetylcholine, have been confirmed to exist in the zebrafish brain [21,55]; however, a better understanding of the comparative brain physiology is required [21].

To obtain a general picture of approximate interspecies similarity of the putative CCAM central targets, we carried out a quantitative alignment analysis of rat (*Rattus norvegicus*), mouse (*Mus musculus*), zebrafish, and human NMDAR subunits, namely, AChE, BChE, and muscarinic acetylcholine receptors, using the NCBI BLAST. 

**Figure 13:**
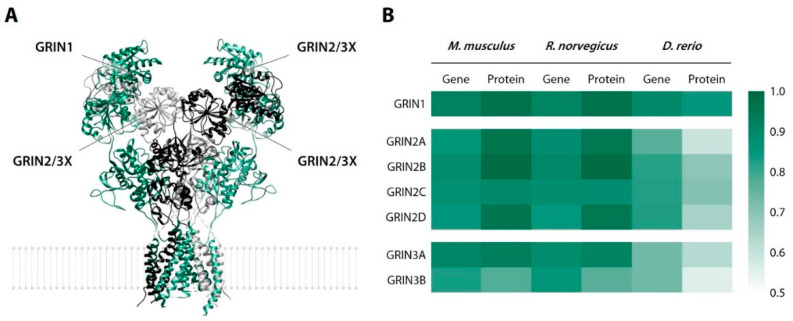
Subunits of N-methyl-D-aspartate receptor as a putative glutamatergic target for chromone-containing allylmorpholines (**A**) and a heatmap of their gene/protein homolog identity to *Homo sapiens* (**B**). GRIN, glutamate receptor, ionotropic (=N-methyl-D-aspartate receptor).

The gene sequence analysis resulted in 82.7–91.6% identity for different NMDAR subunit types (GRIN_1–3_) in *M. musculus*/*R. norvegicus* vs. *H. sapiens*. Amino acid sequences showed a slightly higher mean interspecies similarity, resulting in 77.0–98.7% identity. This apparent discrepancy is a common phenomenon explained by DNA code degeneracy and the presence of introns and/or silent mutations (Figure 13). GRIN_1–3_ genes were 73.4–89.6% identical between *D. rerio* and *H. sapiens*, and they coded for 55.5–84.7% of identical proteins. 

The results we obtained are in agreement with the available data, and they support the hypothesis of the structural and kinetic differences between GLUR types in *H. sapiens* and *D. rerio* being the cause underlining the observed variance in effects of centrally acting drugs. Biperiden and NMDA showed no antagonistic activity towards compound **9a** in Experiments 6 and 9, which appears to be inconsistent with the results of in vitro screening that suggested anticholinergic and NMDA-blocking mechanisms for the CCAMs [13].

The antimuscarinic agent biperiden, similarly to NMDA, showed no antagonism towards the CCAM 9a; moreover, the two agents seemed to act in synergy with each other. This could imply that the CCAMs are, in fact, M-anticholinergics or that their cholinergic activity is complex and cannot be assessed in simple experiments with antagonists. Previously, the AChE blocker donepezil was shown to dose-dependently decrease adult zebrafish locomotor activity and induce an anxiogenic response [56]. Physostigmine, however, exerted an anxiolytic effect, which was blocked but not inverted by scopolamine [57]. Of note, in a recent experiment in larval zebrafish, scopolamine caused AChE inhibition and acted synergistically with the AChE-blocking constituents of *Convolvulus pluricaulis* [58]. 

Such evidence could imply that the zebrafish cholinergic neurotransmission system (and, subsequently, the effects of cholinergic agents) differs anatomically and/or physiologically from that of *H. sapiens*. In vertebrates, cholinergic neurons are present in distinct areas of the brain, including the basal forebrain, the brainstem, and the habenula [59]. It is known that the functional equivalents of the mammalian amygdala, striatum, and hippocampus are represented in the zebrafish brain by the medial zone of the dorsal telencephalon, the dorsal nucleus of the ventral telencephalon [60], and the lateral zone of the dorsal telencephalon, respectively [61,62]. However, the intracellular signaling events that underlie AChE-dependent processes in the zebrafish remain undescribed, and the degree of functional conservation between mammalian and zebrafish brains is unknown [62].

**Figure 14:**
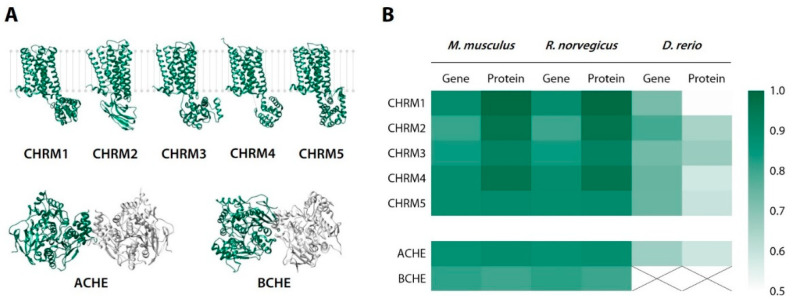
Putative cholinergic targets for chromone-containing allylmorpholines (**A**) and a heatmap of their gene/protein homologue identity to *Homo sapiens* (**B**). CHRM, muscarinic acetylcholine receptor; AChE, acetylcholinesterase; BChE, butyrylcholinesterase.

The BLAST analysis of nucleotide sequences returned 80.2–88.4% identity for the M_1_–M_5_ muscarinic cholinergic receptor subunits (CHRM_1–5_), 86.8–87.0% identity for AChE, and 81.2–82.0% identity for BChE in *M. musculus*/*R. norvegicus* vs. *H. sapiens*. The corresponding protein sequences were 88.7–98.9%, 88.0–88.1%, and 80.0–80.1% identical, respectively (Figure 14). 

The sequence analysis of *H. sapience* vs. *D. rerio*, however, returned 72.7–79.2%, 74.7%, and 66.4% identities for CHRM_1–5_, AChE, and BChE genes, respectively, and 50.6–67.3% identity for the AChE protein. It is known that, compared to humans, key amino acid residues remain conserved within the acyl- and choline-binding sites and the catalytic triad of zebrafish AChE, but evidence suggests its binding and inhibition kinetics may differ across species. BChE, however, is completely absent in zebrafish, which renders this particular test system unsuitable to evaluate BChE-modulating neuroactive agents [22,63].

It must be said that our study has several limitations. First, we were unable to definitively confirm that the observed changes in zebrafish behavior were caused by sedation and not related to the general toxicity of the CCAMs. Second, no data are currently available on the metabolism and pharmacokinetics of the CCAMs, leaving the question of whether the compounds themselves and/or their metabolites can cross the BBB open. Third, we did not employ any molecular methods or techniques to explore the neurochemical background behind the behavioral patterns visually registered.

Further research should be aimed at establishing the dose dependency of pharmacological effects, the pharmacokinetics profile, and the toxicity of the CCAMs in order to determine and distinguish their central effects. Our results suggest a more detailed study of the pharmacological activity of this compound class in rodents and an evaluation of their potential effectiveness in psychopathological conditions while at the same time highlighting possible future directions for the investigation of the molecular mechanisms of the CCAMs.

## 5. Conclusions

In this study, we demonstrated that a novel CCAM class of compounds induce dose-dependent sedation in adult zebrafish. One of the compounds, **33a**, was found to induce an anxiolytic-like response in a lower range of concentrations. CCAM pharmacological activity showed strong dose dependency, allowing for a clear distinction between the anxiolytic-like and sedative effects of low and high doses of compound **4** (**33a**), respectively, a pattern typical of “classical” anxiolytic agents. Taken together, these results imply that the CCAMs may cross the blood–brain barrier and exert central psychotropic effects, which result in alterations in the behavioral phenotype. Therefore, the CCAMs represent an appealing group of molecules and may be of interest as potential anxiolytic, sedative, antipsychotic, general anesthetic, or other psychoactive drug candidates.

In experiments with cholinergic and glutamatergic putative CCAM antagonists, we were unable to confirm the mechanisms of action of the CCAMs suggested by in vitro data. This could potentially result from the specifics of CCAM metabolism or from the interspecies differences between the putative glutamatergic and cholinergic targets. The latter hypothesis is supported by the relatively low identity percentage of their gene and protein sequences, as well as by some unexpected effects of typical agonists and antagonists in our and others’ works.

Our results highlight the undeniable genetic and physiological differences between humans and zebrafish, which does not compromise the translational value of the latter but rather emphasizes the crucial importance of choosing the right model for psychoactive drug screening. Further research in rodents and the use of molecular-based techniques are required to fully explore the pharmacological activity of the CCAMs.

## Figures and Tables

**Table 1:** Chromone-containing allylmorpholines selected for this study and their biological activity [11].

Original Name	Name	Structure	IC_50_, μM; or % Inhibition at 24 μM		
			eqBChE	eeAChE	NMDAR
**9a**	**1**	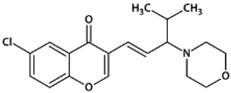	14.50 ± 0.40	15%	28.00 ± 6.00
**9j**	**2**	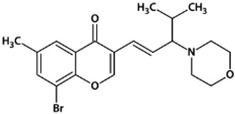	1.20 ± 0.10	7.00 ± 1.00	51.00 ± 12.00
**9l**	**3**	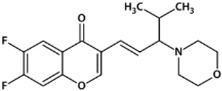	17%	6.40 ± 0.20	nd
**33a**	**4**	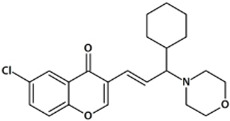	1.60 ± 0.10	14%	15.00 ± 4.00
**33b**	**5**	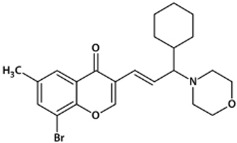	0.42 ± 0.03	7.00 ± 1.00	8.00 ± 3.00

eqBChE, equine butyrylcholinesterase; eeAChE, electric eel acetylcholinesterase; NMDAR, N-methyl-D-aspartate receptor; nd, not determined.

**Table 2:** Working concentrations of all agents used in the experiments.

Experiment	CCAM	Putative Antagonist	Concentrations, mg·L^−1^					
			CCAM			Putative Antagonist		
			a	b	c	a	b	c
1	1	-	1	10	20 ^a^	-		
2	2	-	1	10	100	-		
3	3	-	1	10	100	-		
4	4	-	1	10	100	-		
5	5	-	0.001 ^a^	0.01 ^a^	0.1 ^a^	-		
6	1	NMDA	20			3.83 ^b^		
7	-	NMDA	-			100		
8	-	quinolinic acid	-			10	50	100
9	1	biperiden	20			8.11 ^b^		
10, 11	4	-	0.5	1	5	-		

CCAM, chromone-containing allylmorpholine. ^a^ due to potent activity and/or poor water solubility; ^b^ equimolar to 20 mg·L^−1^ 9a.

## Data Availability

Not applicable.
